# Autophagy Upregulates miR-449a Expression to Suppress Progression of Colorectal Cancer

**DOI:** 10.3389/fonc.2021.738144

**Published:** 2021-10-19

**Authors:** Sheng-Hui Lan, Shu-Ching Lin, Wei-Chen Wang, Yu-Chan Yang, Jenq-Chang Lee, Pei-Wen Lin, Man-Ling Chu, Kai-Ying Lan, Roberto Zuchini, Hsiao-Sheng Liu, Shan-Ying Wu

**Affiliations:** ^1^ Department of Life Sciences and Institute of Genome Sciences, National Yang Ming Chiao Tung University, Taipei, Taiwan; ^2^ Cancer Progression Research Center, National Yang Ming Chiao Tung University, Taipei, Taiwan; ^3^ Department of Microbiology and Immunology, College of Medicine, National Cheng Kung University, Tainan, Taiwan; ^4^ Department of Surgery, College of Medicine, National Cheng Kung University Hospital, Tainan, Taiwan; ^5^ Center for Cancer Research, Graduate Institute of Clinical Medicine, College of Medicine, Kaohsiung Medical University, Kaohsiung, Taiwan; ^6^ Department of Gastroenterology, Hospital Centro Médico, Guatemala City, Guatemala; ^7^ Master of Science Program in Tropical Medicine, College of Medicine, Kaohsiung Medical University, Kaohsiung, Taiwan; ^8^ Department of Microbiology and Immunology, College of Medicine, Taipei Medical University, Taipei, Taiwan; ^9^ Graduate Institute of Medical Sciences, College of Medicine, Taipei Medical University, Taipei, Taiwan

**Keywords:** autophagy, miR-449a, FoxO1, colorectal cancer, tumorigenesis

## Abstract

Many studies reported that microRNAs (miRNAs) target autophagy-related genes to affect carcinogenesis, however, autophagy-deficiency-related miRNA dysfunction in cancer development remains poorly explored. During autophagic progression, we identified miR-449a as the most up-regulated miRNA. MiR-449a expression was low in the tumor parts of CRC patient specimens and inversely correlated with tumor stage and metastasis with the AUC (area under the curve) of 0.899 and 0.736 as well as poor overall survival rate, indicating that miR-449a has the potential to be a prognostic biomarker. In the same group of CRC specimens, low autophagic activity (low Beclin 1 expression and high p62 accumulation) was detected, which was significantly associated with miR-449a expression. Mechanistic studies disclosed that autophagy upregulates miR-449a expression through degradation of the coactivator p300 protein which acetylates the transcription factor Forkhead Box O1 (FoxO1). Unacetylated FoxO1 translocated to the nucleus and bound to the miR-449a promoter to drive gene expression. Either activation of autophagy by the inducer or overexpression of exogenous miR-449a decreases the expression of target gene LEF-1 and cyclin D1, which lead to decreased proliferation, colony formation, migration, and invasion of CRC cells. Autophagy-miR-449a-tartet genes mediated suppression of tumor formation was further confirmed in the xenograft mouse model. In conclusion, this study reveals a novel mechanism wherein autophagy utilizes miR-449a-LEF1-cyclin D1 axis to suppress CRC tumorigenesis. Our findings open a new avenue toward prognosis and treatment of CRC patients by manipulating autophagy-miR-449a axis.

## Introduction

According to 2020 global cancer statistics (GLOBOCAN 2020), colorectal cancer (CRC) is the third in terms of incidence and the second leading cause of cancer-related deaths (1). CRC is influenced by both environmental and genetic factors, which contribute to chronic inflammation and the development of cancer (2). Surgical resection is effective for early-stage CRC patients. However, relapse and metastasis occur after surgery in late-stage CRC patients, and the five-year survival rate of these patients is less than 10%. Moreover, CRC resistant to clinical anticancer drug treatment accounts for 40 to 50% of the poor survival rate (3, 4). Thus, there is an urgent need for more precise diagnostic tools and new strategies to overcome chemoresistance for colon cancer therapy.

Autophagy is a catabolic response against various stresses. The degradation of damaged organelles and proteins provides nutrients and energy to maintain cell survival. Recent reports reveal that autophagy also plays a biogenesis role in protein trafficking and secretion (5, 6). Disruption of autophagy causes various diseases such as neurodegeneration, aging, infection, Crohn’s disease, and cancers (7, 8). Many reports have found evidence indicating that autophagy-related genes (Atg5, Beclin 1, Atg16L1, and UV radiation resistance-associated gene/UVRAG) are either mutated or down-regulated in CRC cells (9–12). Furthermore, accumulating evidence shows that autophagy functions as a transformational switch when a cell shifts from normal to malignant in CRC tumorigenesis (13). However, the mechanism remains elusive. We previously reported that oncogenic miR-224 directly regulated by degradative autophagy machinery promotes hepatocellular carcinoma (14). We also reported that the oncogenic miR-338-5p induces migration, invasion, and metastasis of CRC in part through the PIK3C3-related autophagy pathway, and miR-338-5p/PIK3C3 ratio may become a prognostic biomarker for CRC patients (15). Herein we intend to know whether miRNAs can be regulated by autophagy machinery in CRC.

MicroRNAs act either as a tumor suppressor or as an oncogene in different cancers based on the target genes they suppress (16). Since both autophagy and miRNAs participate in tumorigenesis, their relationship warrants further exploration. Notably, most of the reported miRNAs that inhibit autophagic activity (e.g. miR-101, miR-30a, miR-34a, miR-204, and miR-375) are known tumor suppressors and are downregulated in cancers (17). Many studies focused on the mechanisms by which miRNAs regulate autophagy-related genes, however, very few studies investigate how autophagy regulates miRNAs. In this study, we reveal a novel molecular mechanism that autophagy upregulates the miR-449 to suppress CRC tumorigenesis both *in vitro* and *in vivo*. We further validate the clinical significance in CRC patient specimens.

## Materials and Methods

### Cell Lines and Reagents

SW480 and SW620 cells were cultured in L15 medium (Invitrogen, NY, USA) at 37°C without CO_2_. Colon cancer cells (HCT116 and HT29), prostate cancer cells (PC3), hepatoma cells (Huh7 and Hep3B), lung cancer cells (A549), gastric cancer cells (AGS), and breast cancer cells (MCF-7) were cultured in Dulbecco’s Modified Eagle’s Medium (DMEM) (Invitrogen) at 37°C with 5% CO_2_. All of these cells were supplemented with 10% fetal bovine serum (FBS; Biological Industries, Kibbutz Beit Haemek, Israel) and penicillin/streptomycin (Sigma, MO, USA). Amiodarone, chloroquine (CQ), and FoxO1 inhibitor AS1842856 were purchased from Sigma.

### Tissue Specimens

CRC human specimens were provided by Dr. Jenq-Chang Lee (National Cheng Kung University Hospital, Tainan, Taiwan). Informed consents in accordance with the principles of the Declaration of Helsinki were signed by all the patients with approval by the Institutional Review Board, National Cheng Kung University Hospital, Tainan, Taiwan (IRB document number: B-ER-103-031). The stage of the clinical CRC human specimens was determined according to the Dukes classification. CRC tissue arrays were purchased from the Tissue Bank, National Cheng Kung University Hospital, Taiwan.

### Quantitative Real-Time PCR

Total RNA of the cells was extracted with Trizol™ reagent. One microgram of RNA was reversely transcribed to cDNA using the NCode™ VILO™ cDNA synthesis miRNA kit (Invitrogen). Briefly, the cDNA was synthesized using SuperScript III reverse transcription in the presence of a universal reverse transcription primer (Invitrogen). Real-time PCR was conducted to amplify the cDNA with SYBR Green SuperMix (Invitrogen) using an Applied Biosystems real-time PCR machine. Data were normalized with the internal U54 or β-actin reference control using the comparative C_T_ method.

### Luciferase Reporter Assay

The cells were seeded into the 6-well plates and co-transfected with 1.3 μg reporter plasmid pGL3-basic-0.5K, pGL3-basic-1K, or pGL3-basic-2K with 0.1 μg of the control plasmid (pRL-TK vector) by Lipofectamin^™^ 2000 (Invitrogen). After 48 h, the cell lysate was collected in 100 μl Passive Lysis Buffer. The intensity of fluorescence was determined using the Dual-GIO^®^ Luciferase Assay System following the manufacturer’s instructions (Promega, WI, USA). Each assay was measured in triplicate by a luminometer (Minilumate LB9506, Bad Wildbad, Germany). Luciferase activity of the firefly luciferase was normalized for equal transfection efficiency based on *Renilla* luciferase activity in each lysate, which was used as the internal control.

### Immunohistochemistry

IHC staining of paraffin section slides and tissue array with polyclonal anti-Beclin-1 (Abcam), anti-p62 (Medical and Biological Laboratories), anti-FoxO1 (Cell Signaling, C29H4), anti-Cyclin D1 (Abcam), or anti- LEF-1 (Lymphoid Enhancer Binding Factor 1) (Abcam, ab22884) were performed as previously described (18). Briefly, slides were labeled with biotin-linked secondary antibody followed by Streptavidin (Dako Cytomation, Carpinteria, USA) treatment for 10 min at room temperature. The slides were treated with AEC solution for 10 min and then counterstained with 10% hematoxylin (Muto Pure Chemicals, Tokyo, Japan) and mounted with glycerol gelatin (Sigma).

### MicroRNA *In Situ* Hybridization

MicroRNA ISH was performed as described (14). Briefly, slides were hybridized with digoxigenin (DIG) LNA-modified-miR-449a using the IsHyb *In Situ* Hybridization kit (Biochain, Newark, NJ). Slides were incubated with anti-DIG-HRP antibody for 1 hour. MiR-449a signal was amplified by the TSA Plus DNP system (Perkin Elmer, Waltham, MA) and then incubated with anti-DNP-HRP for 1 hour. Slides were treated with an AEC solution and hematoxylin.

### Chromatin Immunoprecipitation Assay

The cells were seeded onto 10 cm plates and treated with amiodarone (10 μM; Sigma) for 24 h and the ChIP assay was conducted using a ChIP-IT High Sensitivity (HS) kit according to the manufacturer’s instructions (Active Motif, California, USA). Briefly, cells were crosslinked at 37°C for 5 min using 1% formaldehyde. After sonication, the resulting chromatin was diluted 1:10 with ChIP dilution buffer and immunoprecipitated by anti-FoxO1 antibody (Cell Signaling, C29H4) or control IgG. The chromatin-antibody complex was incubated with salmon sperm DNA/Protein A Agarose-50% (Millipore Corp., Billerica, MA, USA) overnight at 4°C with rotation. The DNA was eluted from the beads using ChIP elution buffer and purified by spin column.

### Colony Formation Assay

The six-well plate was layered with 1ml of 0.6% basal agar dissolved in culture medium with serum. One hundred microliters of cell suspension was added to 0.9 ml of 0.4% agar dissolved in culture medium at 37°C. The number of colonies formed was counted after 14 days under a light microscope.

### Mouse Xenograft Tumor Model

Four-week-old female NOD/SCID mice were purchased from the Laboratory Animal Center, National Cheng Kung University (NCKU), College of Medicine, Tainan, Taiwan. The animals were maintained under sterile conditions in laminar flow rooms and provided with sterilized food and water. Animal welfare and the experimental studies complied with United States National Institutes of Health guidelines and the NCKU Laboratory Animal Care and Use Committee Guide for Care and Use of Laboratory Animals. This study was approved by the Institutional Animal Care and Use Committee (IACUC) of National Cheng Kung University (IACUC No: 100074). Eight mice were injected subcutaneously (s. c.) with SW480 cells (1×10^7^/100 μl). After four days the tumors were formed, eight mice were randomly divided into two groups (Control: n=4, Amiodarone: n=4). The amiodarone group was injected intraperitoneally (i.p.) with amiodarone (30 mg/kg). The control group (N.C.) was injected with DMSO. This treatment continued at three-day intervals for a total of 27 days. Tumor volume was measured according to the formula: Volume (mm^3^) = (length × width^2^)/2. The mice were sacrificed at day 27, and the tumors were collected.

### Statistical Analysis

Data are presented as the mean ± SE values (error bars). Differences between the experimental and control groups were analyzed by two-tailed Student’s *t*-test. Receiver operating characteristic (ROC) curves were constructed and the area under the curve (AUC) was calculated to evaluate the specificity and sensitivity of predicting cases and controls. The general guidelines of AUC values are as follows: AUC= 0.5: No discrimination; 0.7≤ AUC < 0.8: Acceptable discrimination; 0.8 ≤ AUC < 0.9: Excellent discrimination; AUC≥ 0.9: Outstanding discrimination. The overall survival rate of CRC patients within 2 years after surgery was generated by Kaplan-Meier survival analysis followed by log-rank test to obtain the p-value.

## Results

### Screening for Autophagy-Related miRNAs Participating in Tumor Formation

In our previous report, we screened autophagy-related miRNAs that participate in tumor formation by miRNA microarray (14), and miR-449a was identified as the lowest expressed miRNAs in the tumors derived from autophagy-deficient cells (**Supplementary Table 1**). Furthermore, we treated the cells with various inducers including Ha-*ras* oncogene (19), starvation (Hank’s balanced salt solution), or rapamycin to induce autophagic activity. Among five autophagy upregulated miRNAs, only miR-449a was ubiquitously upregulated, implying that miR-449a expression is specifically upregulated by autophagy (**Supplementary Table 2**). Low expression of miR-449a in various cancers including prostate, gastric, liver, bladder, and lung cancers has been reported (20). We reveal that colorectal cancer cell line SW480 showed the second lowest level of miR-449a among seven analyzed cancer cell lines (**Supplementary Figure 1**). Similarly, Sun et al, reported that low levels of miR-449a, as well as high level of STAB2 (miR-449 target), promoted CRC development (21). Feng et al, reported that miR-449a was markedly decreased during the neoplastic transformation of ulcerative colitis (UC) to colitis-associated colorectal cancer (CAC) (22). Fu et al., reported that decreased circulating miR-449a is related to poor prognosis in CRC patients (23). These findings support the notion that autophagy-related miR-449a may play a suppressive role in CRC tumorigenesis.

### Low Expression of miR-449a Associated With Low Autophagic Activity Was Detected in Tumors of CRC Patient Specimens

**Figure 1:**
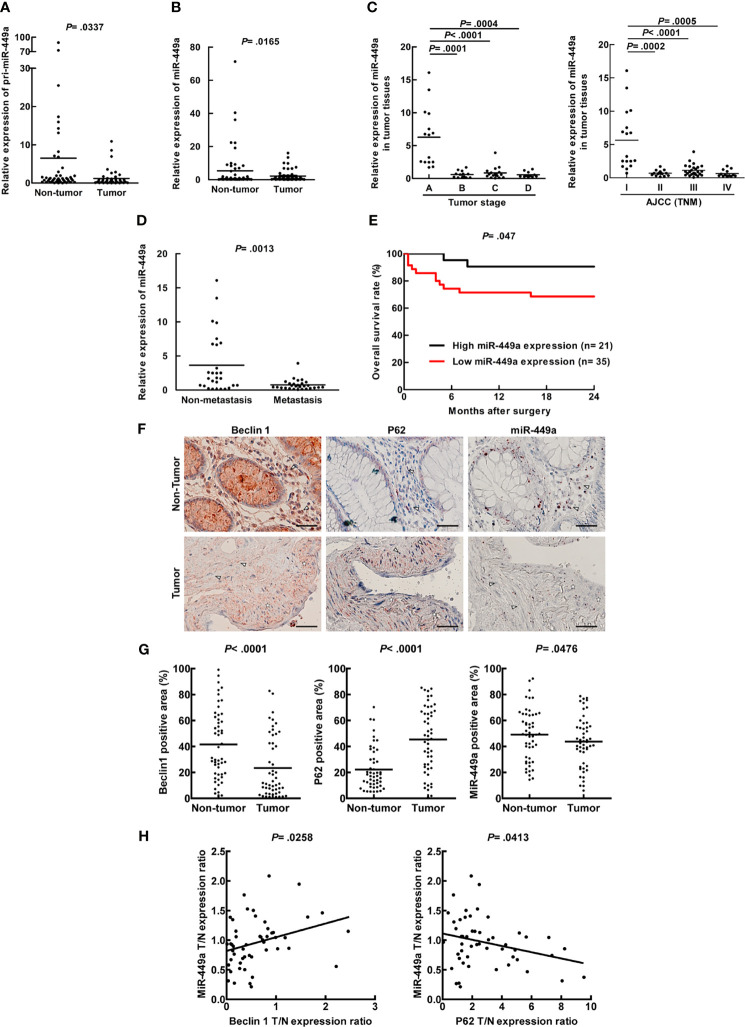
Low expression of miR-449a associated with low autophagic activity was detected in tumors of CRC patient specimens. Real-time PCR was used to analyze the human CRC patient specimens for miR-449a expression levels. **(A)** The expression level of primary miR-449a in 56 paired CRC patient specimens (tumors and adjacent non-tumor parts). **(B)** The expression level of mature miR-449a in above CRC patient specimens. **(C)** The expression of miR-449a in 56 CRC patient specimens grouped by Duke Stages. **(D)** The expression of miR-449a in 56 CRC patient specimens grouped as non-metastasis and metastasis. **(E)** The overall survival rate of CRC patients within 2 years after surgery was generated by Kaplan-Meier survival analysis followed by log-rank test to obtain the p-value. These 56 paired CRC patients were divided into two groups. High miR-449a expression group (n = 21) represents miR-449a expression in the tumor tissue/miR-449a expression in the adjacent non-tumor tissue > 1-fold. Low-miR-449a expression group (n=35) represents miR-449a expression in the tumor tissue/miR-449a expression in the adjacent non-tumor tissue ≤ 1-fold. **(F)** The representative sections from 51 paired CRC specimens in the tissue array showing the expression levels of Beclin 1, p62, and miR-449a. MiR-449a level was shown by *in situ* hybridization labeling miRNA. Scale bar= 50 μm. △ represents the stromal area and ☆ represents the tumor area. **(G)** Beclin 1, p62, and miR-449a levels were quantified by defining regions of interest (ROIs) using automated cell acquisition and quantification software (Histoquest). **(H)** Correlation of miR-449a expression with Beclin 1 and p62 was analyzed from the 51 paired CRC specimens in the tissue array and linear regression coefficient and statistical significance are indicated.

To confirm the significance of miR-449a in CRC patient specimens, a total of 56 CRC patient specimens were analyzed (**Supplementary Table 3**) to determine miR-449a expression levels in tumors and adjacent non-tumor parts. We found that both primary and mature miR-449a RNA expression levels were low in the tumors compared with the adjacent non-tumor parts by real-time PCR analysis (**Figures 1A, B**). Moreover, miR-449a expression was much higher in stage A specimens compared to other stages in the Dukes staging system (**Figure 1C**; stage A *vs*. stage B, C, and D). We also analyzed miR-449a expression by AJCC (TNM) staging system, and our data showed that miR-449a expression was higher in stage I compared to other stages (**Figure 1C**; TNM stage I *vs*. stage II, III, and IV). These two staging systems showed similar trends of miR-449a expression in CRC patient specimens. Furthermore, miR-449a expression was low in metastatic tumors compared to the non-metastatic tumors (**Figure 1D**). We conducted a receiver operating characteristics (ROC) curve analysis to evaluate whether miR-449a has the potential to be a biomarker for prognosis. Our data showed that the AUC (area under the curve) values for tumor stage and metastasis were 0.899 and 0.736, respectively (**Supplementary Figure 2**), indicating that miR-449a is a potential marker for these two events. We conducted Kaplan-Meier survival analysis followed by log-rank test to correlate miR-449a expression with CRC patient survival rate. Our result showed that low miR-449a expression correlated with poor overall survival rate among 56 CRC patients (**Figure 1E**). In summary, miR-449a expression is inversely correlated with CRC tumorigenesis and is a potential diagnostic or prognostic biomarker for CRC patients.

Accumulating reports demonstrated that autophagic activity was determined by the protein expression of autophagy-related proteins including Beclin 1 (*ATG6*) and p62 (*SQSTM1)*; therefore, we conducted the CRC tissue array to clarify the relationship between autophagic activity and miR-449a. A total of 51 paired CRC specimens (tumor and adjacent non-tumor tissue) in the tissue array were analyzed (**Supplementary Table 4**). The protein expression of Beclin 1 and p62 was evaluated by IHC, and miR-449a RNA expression was determined by miRNA *in situ* hybridization (ISH) assay. We further quantitatively analyzed the IHC data by HistoQuest Image Analysis Software. Our results showed low Beclin 1 level, high p62 accumulation as well as low miR-449a level in the tumors compared with the adjacent non-tumor tissues (**Figures 1F, G**), which is consistent with the results of miR-449a by real-time PCR (**Figures 1A, B**). Furthermore, the *in silico* CPTAC sample analysis showed low Beclin 1 protein expression in the tumor parts compared to the non-tumor parts (**Supplementary Figure 3**). Moreover, miR-449a level was positively correlated with Beclin 1 expression and negatively correlated with p62 accumulation in the 51 CRC specimens (**Figure 1H**). Altogether, low miR-449a expression correlated with decreased autophagic activity in CRC patient specimens.

### Autophagy Positively Regulates miR-449a Expression at the Transcriptional Level in CRC Cells

**Figure 2:**
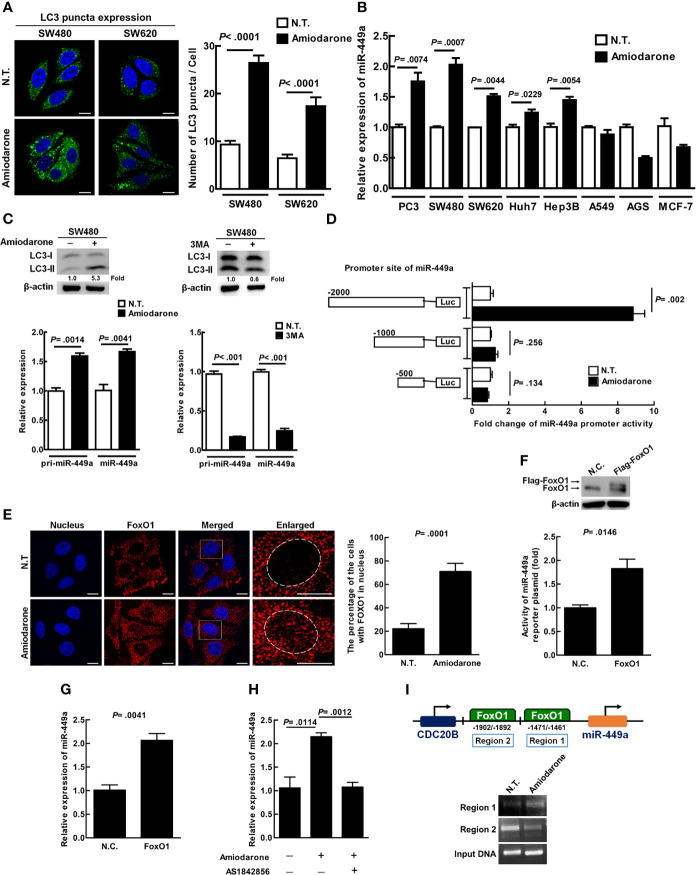
Autophagy positively regulates miR-449a expression at the transcriptional level through FoxO1 binding at region 1 of the promoter in CRC cells. **(A)** SW480 and SW620 cells were treated with amiodarone (10 μM) for 48 h. Anti-LC3 antibody and Hoechst stain were used to label the treated cells for LC3 puncta and nuclei, respectively. Cells were investigated under a confocal microscope. Quantification of LC3 puncta was conducted by randomly counting 30 cells. The data represent means ± SEM. **(B)** The expression of miR-449a was determined by real-time RT–PCR under amiodarone (10 μM) treatment for 48h in colorectal (SW480 and SW620), prostate (PC3), liver (Huh7 and Hep3B), breast (MCF-7), lung (A549), and gastric (AGS) cells. U54 was used as an endogenous control to normalize miR-449a expression. **(C)** SW480 cells were treated with amiodarone (10 μM) for 48 h or treated with the inhibitor 3MA (10 mM) for 24 h followed by immunoblotting to determine LC3 protein level. The levels of pri-miR-449a and miR-449a in SW480 cells were measured by real-time PCR. **(D)** Schematic diagram shows the miR-449a reporter plasmids constructed. The cells were transiently transfected with the constructed plasmid pGL3-basic-500, 1000 or 2000 bp. The relative luciferase activities were measured after normalization with *renilla* luciferase activity with or without amiodarone (10 μM) treatment for 48 h. **(E)** Localization of FoxO1 and nucleus was shown using anti-FoxO1 antibody and Hoechst staining, respectively. The cells were investigated under a multi-photon confocal microscope and quantified by randomly counting 50 cells. **(F)** SW480 cells were transiently transfected with FoxO1 plasmid (4 μg, pcDNA3/FLAG-FoxO1) by Lipofectamine™ 2000 for 48 h. The expression of FoxO1 was measured using anti-FoxO1 antibody by immunoblotting. The activity of miR-449 reporter plasmid was measured by luciferase assay. **(G)** The expression of miR-449a was measured after the cells were transiently transfected with FoxO1 plasmid DNA (8 μg) for 48 h by real-time PCR. **(H)** The cells were treated with amiodarone (10 μM) in the presence or absence of FoxO1 inhibitor AS1842856 (40 nM) for 48 h. The expression of miR-449a was determined by real-time PCR. **(I)** SW480 cells after amiodarone (10 μM) treatment for 24 h were fixed with formaldehyde followed by immunoprecipitation with anti-FoxO1 antibody. The DNA in the immunoprecipitate was amplified using the primer sets encompassing region 1 and 2 of miR-449a promoter region. Student’s *t*-test analysis was conducted and p-value ≤ 0.05 represents significance.

Amiodarone, the mTOR inhibitor, induces autophagy and sustains longer autophagic activity after treatment as compared to other autophagy inducers including rapamycin (24). We demonstrated that amiodarone treatment increased LC3 puncta in SW480 and SW620 cells (**Figure 2A**), indicating amiodarone increased autophagic activity in cells. Furthermore, we reveal that colorectal cancer cell line SW480 showed a higher level of miR-449a under autophagy induction among eight analyzed cancer cell lines (**Figure 2B**). We demonstrated that both pri-miR-449a and mature miR-449a expression were significantly increased under autophagy induction conditions which LC3-II expression increased (**Figure 2C**, upper left panel), by real-time PCR analysis (**Figure 2C**, lower left panel). Accordingly, autophagy inhibitor 3-Methyladenine (3MA) treatment decreased LC3-II expression (**Figure 2C**, upper right panel) accompanied with decreased pri-miR-449a and miR-449a expression (**Figure 2C**, lower right panel). These results correlated with decreased expression of pri-miR-449a and miR-449a in CRC tumor specimens. These findings imply that autophagy positively regulates miR-449a expression at the transcriptional level in CRC cells and tumors.

### Autophagy Increases miR-449a Expression Through FoxO1 Binding at the Promoter Region of miR-449a

CDC20B is the host gene for miR-449a transcription. We clarified whether autophagy upregulates miR-449a through the regulation of CDC20B gene in SW480 cells. Our data showed that CDC20B gene expression was not induced after autophagy induction by amiodarone for 48 h (**Supplementary Figure 4A**), indicating that autophagy did not regulate miR-449a through the CDC20B host gene. We then measured miR-449a promoter activity by cloning three promoter regions, 500 base-pair (bp), 1000 bp, and 2000 bp, upstream of the miR-449a transcription starting site in the luciferase reporter plasmid (pGL3-Basic), and found that the luciferase activity of 2000 bp miR-449a luciferase reporter plasmid increased about 10-fold after autophagy induction (**Figure 2D**), suggesting that autophagy increases miR-449a activity within the promoter region of 1000 to 2000 bp. Transcription factor FoxO1 has been demonstrated to be involved in cellular mechanisms including cell proliferation, differentiation, apoptosis, and autophagy (25). We predicted two FoxO1 binding sites within the region of 1000 to 2000 bp upstream of the miR-449a transcriptional starting site using the TFSEARCH software program. We also detected increased FoxO1 protein translocation from cytoplasm to nucleus when autophagy was induced (**Figure 2E**). We further overexpressed the FoxO1 gene in SW480 cells (**Figure 2F**, upper panel), and detected increased miR-449a promoter activity (**Figure 2F**, lower panel) and miR-449a RNA expression (**Figure 2G**). Accordingly, autophagy induced miR-449a promoter activity and RNA expression were suppressed by FoxO1 inhibitor (AS1842856) (**Supplementary Figure 4B** and **Figure 2H**). Moreover, we confirmed that FoxO1 robustly interacts with region 1 of the miR-449a promoter (-1461~-1471 bp of miR-449a gene) in the presence of amiodarone by ChIP assay (**Figure 2I**). Altogether, our data imply that after autophagy induction, FoxO1 is translocated to the nucleus and bound to the promoter to drive miR-449a expression.

### Amiodarone-Induced Autophagic Machinery Degrades p300 Protein and Activates FoxO1

**Figure 3:**
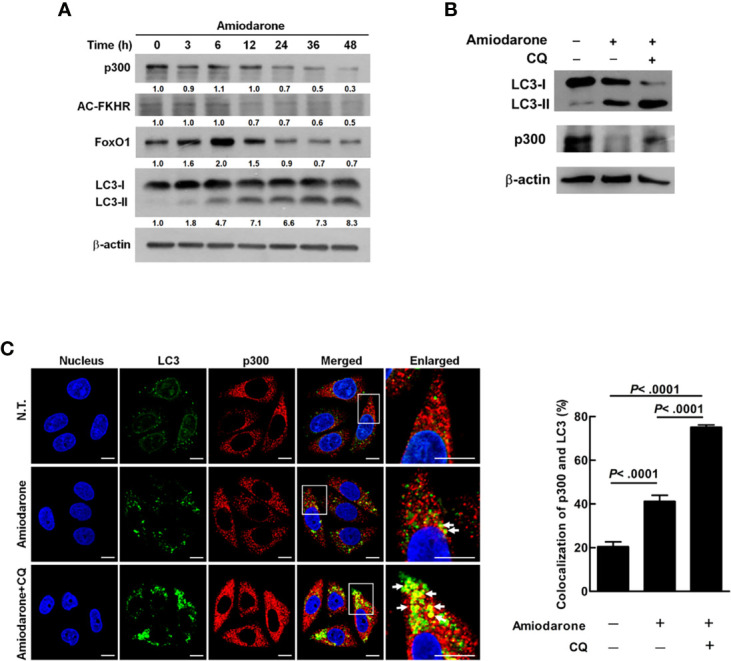
Amiodarone-induced autophagic machinery degrades p300 protein, activates FoxO1, and increases miR-449a expression. **(A)** SW480 cells were treated with amiodarone (10 μM) for 48 h. The levels of p300, AC-FKHR, FoxO1, and LC3 were measured using anti-p300, anti-AC-FKHR, anti-FoxO1, and anti-LC3 antibody by immunoblotting. β-actin was used as the internal control. **(B)** Cells were treated with amiodarone (10 μM) for 48 h. In the CQ group, the cells were treated with amiodarone (10 μM) together with CQ (50 μM) for 48 h. The p300 and LC3 levels were determined by immunoblotting using specific antibodies. β-actin was used as the internal control. **(C)** Colocalization of p300 and LC3 proteins were demonstrated by anti-p300 conjugated with rodamin (red) and anti-LC3 conjugated with FITC (green) antibody, respectively. The fluorescent change of the cells was investigated under a multi-photon confocal microscope. Quantification of colocalization was performed by counting 30 cells. The *p*-values were determined by Student’s *t*-test analysis.

FoxO1 acetylation by p300 histone acetyltransferase (HAT) blocks its translocation to the nucleus as well as DNA-binding capability; whereas unacetylated FoxO1 moves from the cytoplasm to the nucleus and binds to the target gene for transcription (26). Our data showed that amiodarone treatment gradually increased LC3-II expression together with decreased expression of p300, total FoxO1, as well as acetylated FoxO1 (AC-FKHR) proteins in a time-dependent manner (**Figure 3A**). It has been reported that p300 is ubiquitinated followed by recruitment and degradation of the inclusion body by the autophagic machinery in the cytoplasm (27, 28). To clarify whether p300 is an autophagic degradation substrate, chloroquine (CQ, an inhibitor of lysosomal acidification and fusion of lysosome and autophagosome) was used to block lysosome-autophagosome fusion and degradation. Our results demonstrated LC3-II accumulation in the presence of CQ for 48 h, indicating the autophagy degradation process is effectively blocked. Moreover, autophagy induction suppressed p300 expression was reversed by CQ treatment (**Figure 3B**, lane 2 *vs*. lane 3). Similarly, amiodarone treatment increased colocalization of p300 and LC3 puncta (autophagosomes), and the percentage of LC3 and p300 colocalization was further increased in the presence of CQ for 24 h (**Figure 3C**). Altogether, autophagic machinery degrades p300 protein, which in turn affects the expression, acetylation, and function of FoxO1.

### MiR-449a Suppresses Tumorigenesis of CRC Cells *In Vitro*

**Figure 4:**
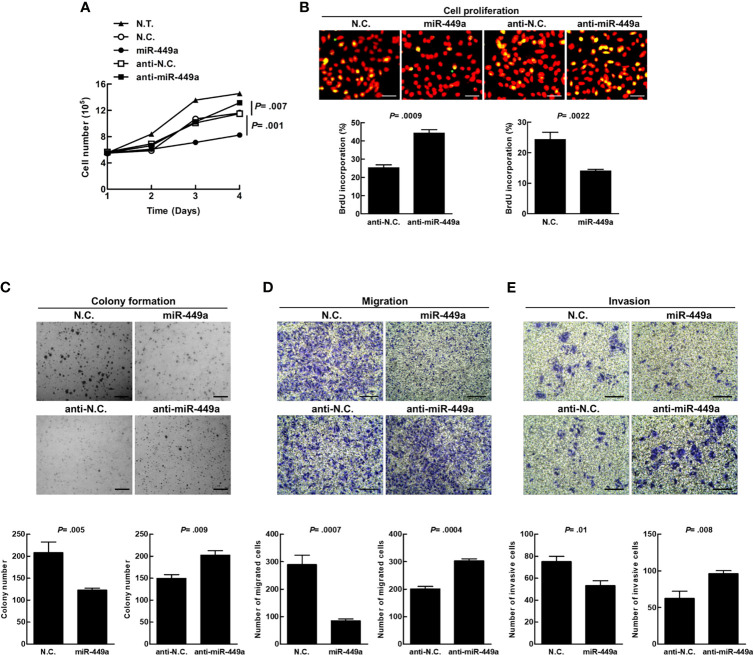
MiR-449a suppresses tumorigenesis of CRC cells. The SW480 cells were transiently transfected with 100 nM of miR-449a, anti-miR-449a, or scramble control (anti-N.C.) for 48 h using Lipofectamine™ 2000 reagent. **(A)** Cell number was counted under a light microscope. **(B)** DNA synthesis was determined by BrdU (0.01 g/ml) incorporation treatment of SW480 cells for 30 min. Anti-BrdU antibody and propidium iodide (PI) were used to label the treated cells for DNA synthesis and nuclei, respectively. The yellow fluorescent cells represent proliferating cells (DNA synthesis), which were counted under three random fields under a fluorescent microscope and were used to calculate the percentage of cell proliferation. **(C)** A total of 2×10^4^ transfected cells were plated on the soft agar in a 6-well plate. The images were taken 14 days after plating. The diameter of a counted colony bigger than 0.1 mm at 9X magnification was defined as positive colony. Scale bar= 1 mm. **(D)** In the migration assay, transfected cells were seeded in the Transwell™ permeable insert with FBS-free medium in the lower chamber. The migrated cells were counted after 48 h. **(E)** In the invasion assay, the transfected cells were seeded into the Transwell™ insert coated with the matrigel. The number of invasive cells was counted after 72 h. The cells were counted under three random fields under a light microscope. Quantification was conducted based on three independent experiments. The *p* values were determined by Student’s *t*-test analysis. Scale bar= 100 μm.

MiR-449a has been reported to suppress cell growth, migration, and invasion of diverse cancer cells (29, 30). We transiently transfected mature miR-449a or antagonist miR-449a (anti-miR-449a) into SW480 cells. Overexpression of exogenous miR-449a suppressed cell growth (**Figure 4A**), BrdU incorporation (**Figure 4B**), colony formation (**Figure 4C**), migration (**Figure 4D**), and invasion (**Figure 4E**), whereas overexpression of anti-miR-449a reversed the effects of miR-449a on the cells (**Figures 4A–E**). These data imply that miR-449a plays a suppressive role in CRC tumorigenesis *in vitro*.

### MiR-449a Targets and Suppresses Cyclin D1 and LEF-1 Expression at Translation Level

Cyclin D1 and LEF-1 (Lymphoid Enhancer Binding Factor 1) are overexpressed in colon cancer and have been identified as the target genes of miR-449a in human prostate cancer and mesenchymal stem cells (31, 32). We transfected miR-449a or anti-miR-449a into SW480 and SW620 cells, and the overexpressed miR-449a significantly suppressed cyclin D1 and LEF-1 protein expression, but the overexpressed antagonist miR-449a (anti-miR-449a) reversed this suppressive effect (**Supplementary Figure 5A**). However, under such conditions, no change was detected in gene expression of Cyclin D1 and LEF1 (**Supplementary Figures 5B, C**). Moreover, LEF-1 and/or cyclin D1 expression was decreased by amiodarone treatment in a dose-dependent manner in SW480, SW620, HCT116, and HT29 cells (**Supplementary Figure 5D**). Altogether, these results imply that autophagy-induced miR-449a specifically targets and suppresses cyclin D1 and LEF-1 genes at the translational level.

### Amiodarone-Induced miR-449a Specifically Targets LEF-1 and Cyclin D1 Translation and Suppresses CRC Tumorigenesis Both *In Vitro* and *In Vivo*

Functional analysis in CRC cell line model reveals that amiodarone-induced autophagy suppressed cell growth, BrdU incorporation, colony formation, cell migration, and invasion (**Supplementary Figures 6A–E**). In summary, amiodarone treatment or overexpression of miR-449a showed a similar effect on colorectal cancer tumorigenesis, supporting the notion that autophagic activation suppresses colorectal cancer tumorigenesis through upregulation of miR-449a.

**Figure 5:**
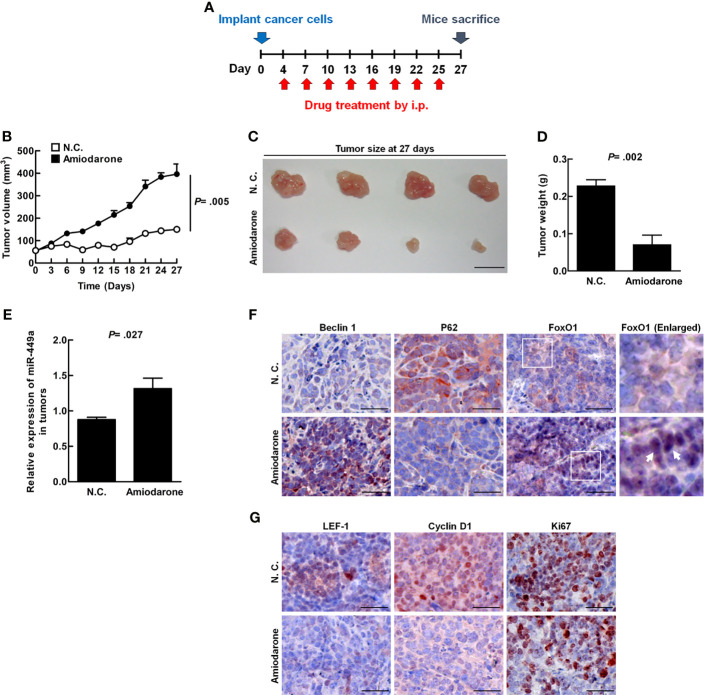
Amiodarone-induced miR-449a specifically blocks LEF-1 and cyclin D1 translation and suppresses CRC tumorigenesis *in vivo.* The four-week-old female NOD/SCID mice (n = 4) were s. c injected with SW480 cells (1x10^7^ cells/100 μl). The mice were then injected intraperitoneally with 30 mg/kg amiodarone after four days’ inoculation every 3 days for 27 days. **(A)** A schematic design of cancer cell inoculation and amiodarone injection in the animal experiment. **(B)** The tumor volume measured at 3-day intervals until day 27. **(C)** After mice were sacrificed, the tumors were collected and photographed. **(D)** Tumor weights were measured. **(E)** The expression of miR-449a in the tumors was determined by real-time PCR. **(F)** The protein levels (red color) of Beclin 1, p62, and FoxO1 in the tumors with or without amiodarone treatment was determined by specific antibodies using IHC staining. The arrow points the FoxO1 staining in the nucleus. **(G)** The levels of LEF-1, cyclin D1, and Ki67 in the tumors with or without amiodarone treatment were determined by IHC staining. The *p* values were determined by Student’s *t*-test analysis.

**Figure 6:**
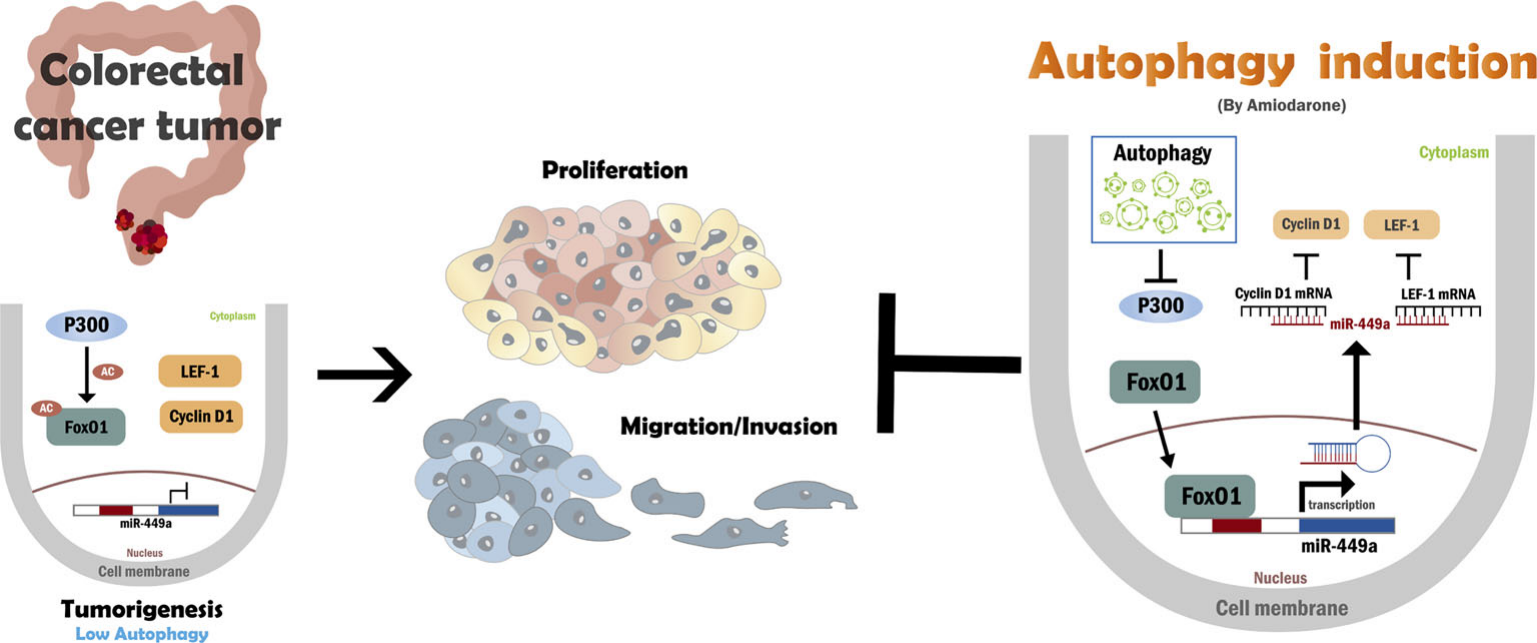
A schematic hypothesis of autophagy regulates miR-449a, LEF-1, Cyclin D1, p300, and FoxO1 during CRC development. In colorectal cancer, low autophagic activity leads to p300 overexpression and increased FoxO1 acetylation. Acetylated-FoxO1 remains in the cytoplasm and prevents its transcriptional activation of miR-449a. Subsequently, the proteins of miR-449a target genes LEF-1 and cyclinD1 remain at high levels, which are responsible for the promotion of CRC tumorigenesis. Autophagy induction by amiodarone degrades p300 protein expression and promotes FoxO1 translocation into the nucleus due to lack of acetylation of FoxO1. MiR-449a expression was then increased by FoxO1, which further suppresses LEF-1 and cyclin D1 expression and leads to suppression of colorectal cancer tumorigenesis.

The xenograft mouse tumor model was established to clarify whether autophagy plays a suppressive role in CRC tumor formation *in vivo*. Four days after SW480 cell inoculation followed by amiodarone treatment at a three day intervals until day 27 (**Figure 5A**), tumor volume, size, and weight were dramatically reduced in the amiodarone group (Amiodarone) compared to the control group (N.C.) at day 27 (**Figures 5B–D**). The levels of miR-449a increased in the tumors of the amiodarone group compared to the N.C. group by real-time PCR analysis (**Figure 5E**). We further detected high Beclin 1 expression, low p62 accumulation (representing high autophagy activation), and increased FoxO1 staining in the tumors as well as in the nucleus of tumor tissues in amiodarone treatment group (**Figure 5F**). Moreover, the levels of LEF-1, cyclin D1, and ki67 (cell proliferation marker) in the tumors with amiodarone treatment group were low compared to the tumors without treatment (N.C.) (**Figure 5G**). Altogether, our results support the notion that amiodarone induces autophagic activity, which leads to FoxO1 translocation to the nucleus to promote miR-449a expression and suppresses cell proliferation through silencing the expression of cyclin D1 and LEF-1 (**Figure 6**).

### Clinical Relevance of FoxO1, LEF-1, and cyclinD1 in CRC Patient Specimens on the Tissue Array and TCGA Data Analysis

Finally, we used 18 paired CRC patient specimens (**Supplementary Table 5**) in the CRC tissue array to demonstrate that the expression of FoxO1 in the nuclei was significantly lower in tumor specimen and the miR-449a target genes LEF-1 was highly expressed in the tumor parts compared to the non-tumor parts (**Supplementary Figure 7**). Similarly, Clinical Proteomic Tumor Analysis Consortium (CPTAC) big data analysis [website http://ualcan.path.uab.edu/index.html (33)] indicates that high mRNA levels of cyclin D1 and LEF-1 in CRC tumor parts compared to non-tumor specimens (**Supplementary Figures 8A, B**). In addition, higher LEF-1 expression correlates with poor overall CRC patient survival rate (**Supplementary Figure 8C**). Altogether, the findings of the CRC patient specimens are consistent with the investigation in our cell line and xenograft mouse model. This conclusion was further supported by the TCGA CRC dataset analysis.

## Discussion

In this study, we demonstrated that autophagy positively regulates miR-449a expression through degradation of coactivator p300 followed by translocation of unacetylated FoxO1 from the cytoplasm to nuclei to bind with miR-449a promoter and drive gene expression. Upregulated miR-449a suppresses CRC tumorigenesis including cell proliferation, migration, invasion, and tumor formation by silencing the expression of target genes Cyclin D1 and LEF-1 at the translational level (**Figure 6**). We reveal that miR-449a expression was low in CRC patient tumors and highly associated with CRC tumor stage and metastasis based on the high AUC values, indicating that miR-449a has the potential to be a prognosis marker of CRC tumor stage and metastasis. This conclusion was consistent with others’ findings (22, 34).

MiR-449a has been predicted to be transcribed within its host gene, CDC20B, by chromatin structure analysis (35). However, CDC20B mRNA level could not be induced under autophagic induction conditions (**Supplementary Figure 4A**), suggesting that autophagy upregulates miR-449a through its proximal promoter. We confirmed that FoxO1 preferentially binds to the 1461~-1471 bp region of miR-449a promoter by ChIP assay under autophagy induction conditions. Similarly, Jitrapakdee et al. reported that FoxO1 translocated from cytoplasm into the nucleus to increase the transcription of gluconeogenesis enzymes under starvation conditions (36). Moreover, cytosolic FoxO1 not only induces autophagic activity but also acts as a tumor suppressor in colon tumors (37). In addition, FoxO1 functions as a transcription factor in the nucleus to regulate the expression of target genes including miR-449a during autophagy induction.

The histone acetyltransferase protein p300, a coactivator family member, interacts with diverse transcription factors and increases the target gene expression. FoxO1 is acetylated by p300, which further blocks FoxO1 cytoplasm-nucleus translocation, promoter binding, and transcriptional capabilities (38). Ishihama et al. reported that p300 was overexpressed in colorectal cancer and is an indicator of poor prognosis in CRC patients (39). Our findings reveal that activated autophagy increases p300 protein degradation and results in decreased FoxO1 acetylation (**Figures 3A, B**), increased FoxO1 translocation to the nucleus (**Figure 2E**) to drive target gene expression (including miR-449a). It is known that the protein harboring LIR (LC3-interacting region) motif may directly interact with the LC3 protein on the autophagosome; however, p300 has no LIR motif according to an analysis by iLIR software (40). Therefore, how p300 was recruited by the autophagosome remains unknown. Chen et al. reported that ubiquitinated p300 protein was recruited by the cytoplasmic inclusion bodies and degraded through the 26S proteasome pathway (27). In addition, cytoplasmic inclusion bodies were degraded through autophagic machinery during liver injury and neurodegeneration (28). Therefore, proteasomal and autophagic degradation systems are two major types of machinery in the degradation of ubiquitinated proteins. Based on these findings, it is probable that p300 may be degraded through autophagic degradation machinery by ubiquitination. Further study is required to clarify the role of autophagy in p300 degradation.

Overexpression of miR-449a significantly decreased CRC cell tumorigenesis and suppressed the expression levels of cyclin D1 and LEF-1 in CRC cells. Cyclin D1 is a regulator of cell cycle progression, controls cell proliferation, and is highly expressed in various human cancers including CRC (41). We previously reported that autophagy directly degrades ubiquitinated-cyclin D1 mediated by p62 in HCC (42, 43). Here, we reveal that autophagy decreases cyclin D1 expression through miR-449a targeting in CRC, suggesting that autophagy regulates cyclin D1 expression either by degradation process or by miRNA targeting. LEF-1 cross-talks between HGF/c-Met and Wnt/β-catenin to coordinate tumor migration and invasion (44). Furthermore, LEF-1 is a prognostic biomarker of liver metastasis in CRC (45). We demonstrated herein that overexpression of miR-449a suppressed the expression of cyclin D1 and LEF-1 at the translational level (**Supplementary Figure 5**). In the xenograft mouse model, amiodarone-induced autophagy increased miR-449a expression together with increased Beclin 1, FoxO1 expression, and decreased p62 accumulation as well as cyclin D1, LEF-1, and Ki67 expression (**Figures 5F, G**). Subsequently, SW480 cell-induced tumor size, volume, and weight were decreased when autophagy was induced by amiodarone (**Figures 5B–D**). We further used CRC patient specimens to validate the significance of autophagy-regulated miR-449a through the FoxO1-LEF-1-cyclin D1 axis in CRC tumorigenesis (**Supplementary Figure 7**). These findings are consistent with our *in vitro* and *in vivo* investigation and sustained by the TCGA CRC dataset analysis. Sun et al. reported that the low level of miR-449a and high level of SATB2 promote CRC development (21). Feng et al. reported that that miR-449a inhibited the growth and metastasis of CRC cells by directly binding to the 3’−UTR of Notch-1 and thereby, suppressed the activation of the Notch signaling pathway (22). In addition to the above mechanisms, we reveal a novel mechanism that autophagy degrades p300, which leads to translocation of FoxO1 to upregulate miR-449a followed by downregulation of two target genes LEF-1 and cyclinD1 in CRC tumorigenesis.

We previously reported that low autophagic activity resulted in high oncogenic miR-224 expression which leads to the development of hepatocellular carcinoma (14). In contrast, this report reveals that low autophagic activity downregulates miR-449a expression participating in colorectal cancer tumorigenesis. Similarly, low miR-449a expression was correlated with poor disease-free survival and histological scores (34), and has been reported during the progression of neoplastic transformation of ulcerative colitis (UC) to colitis-associated colorectal cancer (CAC) (22) as well as in HCC (46) and miR-224 overexpression has also been reported in inflammatory bowel disease-associated CRC (47). Autophagy probably suppresses tumorigenesis through either downregulation of miR-224 or upregulation of miR-449a depending on the types of cancers. Further study is needed to clarify this possibility. Altogether, our novel findings warrant the exploration of novel autophagy-related therapy for the prevention and treatment of CRC.

## Data Availability Statement

The original contributions presented in the study are included in the article/**Supplementary Material**. Further inquiries can be directed to the corresponding authors.

## Ethics Statement

The studies involving human participants were reviewed and approved by the Institutional Review Board, National Cheng Kung University Hospital, Tainan, Taiwan (IRB document number: B-ER-103-031). Written informed consent for participation was not required for this study in accordance with the national legislation and the institutional requirements. The animal study was reviewed and approved by the Institutional Animal Care and Use Committee (IACUC) of National Cheng Kung University (IACUC No: 100074).

## Author Contributions

S-HL contributed to the design, implementation of the research, clinical study analyses, and validation. S-CL, W-CW, and Y-CY performed the experiments. J-CL conducted clinical study analyses. P-WL and M-LC conducted TCGA data analysis. K-YL conducted the scientific illustration. RZ provide clinical advice and contributed to the manuscript editing. H-SL contributed to the design, supervision, and manuscript editing. S-YW wrote the original manuscript, devised the project, the main conceptual ideas, and a proof outline. S-HL, H-SL, and S-YW have verified the underlying data. All authors contributed to the article and approved the submitted version.

## Funding

This study was supported by the grants from the Ministry of Science and Technology, Taiwan, R.O.C. (MOST 109-2314-B-038-119-MY2 to S-YW; MOST 104-2320-B-006-021-MY3 to H-SL), Taipei Medical University (TMU108-AE1-B39 to S-YW), Ministry of Health and Welfare (MOHW 106-TDU-B-211-124-003 to H-SL) and Kaohsiung Medical University Research Center Grant (KMU-TC108A04-0, KMU-TC108A04-2, and KMU-TC109A04-1 to H-SL).

## Conflict of Interest

The authors declare that the research was conducted in the absence of any commercial or financial relationships that could be construed as a potential conflict of interest.

## Publisher’s Note

All claims expressed in this article are solely those of the authors and do not necessarily represent those of their affiliated organizations, or those of the publisher, the editors and the reviewers. Any product that may be evaluated in this article, or claim that may be made by its manufacturer, is not guaranteed or endorsed by the publisher.

## Abbreviations

3MA, 3-Methyladenine; AUC, Area under the curve; ATG, Autophagy-related genes; CAC, Colitis−associated colorectal cancer; CRC, Colorectal cancer; ChIP, Chromatin immunoprecipitation; CQ, Chloroquine; FoxO1, Forkhead Box O1; IHC, Immunohistochemistry; i.p., Intraperitoneally; LEF-1, Lymphoid Enhancer Binding Factor 1; miRNA, microRNAs; miRNA ISH, miRNA *In Situ* Hybridization; ROC, Receiver operating characteristic; Real-time PCR, Real-time polymerase chain reaction; s.c., Subcutaneously; UC, Ulcerative colitis.

## References

[1] Sung H Ferlay J Siegel RL Laversanne M Soerjomataram I Jemal A. Global Cancer Statistics 2020: GLOBOCAN Estimates of Incidence and Mortality Worldwide for 36 Cancers in 185 Countries. CA Cancer J Clin (2021) 71(3):209–49. doi: 10.3322/caac.21660 33538338

[2] Li H Zhao L Lau YS Zhang C Han R. Genome-Wide CRISPR Screen Identifies LGALS2 as an Oxidative Stress-Responsive Gene With an Inhibitory Function on Colon Tumor Growth. Oncogene (2021) 40(1):177–88. doi: 10.1038/s41388-020-01523-5 PMC7790754 33110234

[3] McFadyen MC Melvin WT Murray GI. Cytochrome P450 Enzymes: Novel Options for Cancer Therapeutics. Mol Cancer Ther (2004) 3(3):363–71. 15026557

[4] Zheng X Liu J Li X Tian R Shang K Dong X. Angiogenesis Is Promoted by Exosomal DPP4 Derived From 5-Fluorouracil-Resistant Colon Cancer Cells. Cancer Lett (2021) 497:190–201. doi: 10.1016/j.canlet.2020.10.009 33039561

[5] Deretic V Jiang S Dupont N. Autophagy Intersections With Conventional and Unconventional Secretion in Tissue Development, Remodeling and Inflammation [Research Support, American Recovery and Reinvestment Act, Research Support, N.I.H., Extramural, Research Support, Non-U.S. Gov't, Review]. Trends Cell Biol (2012) 22(8):397–406. doi: 10.1016/j.tcb.2012.04.008 22677446 PMC3408825

[6] Kim YH Kwak MS Lee B Shin JM Aum S Park IH. Secretory Autophagy Machinery and Vesicular Trafficking Are Involved in HMGB1 Secretion. Autophagy (2020) 5:1–18. doi: 10.1080/15548627.2020.1826690 PMC8496717 33017561

[7] Saha S Panigrahi DP Patil S Bhutia SK. Autophagy in Health and Disease: A Comprehensive Review. BioMed Pharmacother (2018) 104:485–95. doi: 10.1016/j.biopha.2018.05.007 29800913

[8] Rana T Behl T Sehgal A Mehta V Singh S Bhatia S. Exploring the Role of Autophagy Dysfunction in Neurodegenerative Disorders. Mol Neurobiol (2021) 58(10):4886–905. doi: 10.1007/s12035-021-02472-0 34212304

[9] Choi JH Cho YS Ko YH Hong SU Park JH Lee MA. Absence of Autophagy-Related Proteins Expression Is Associated With Poor Prognosis in Patients With Colorectal Adenocarcinoma. Gastroenterol Res Pract (2014) 2014:179586. doi: 10.1155/2014/179586 24723943 PMC3960741

[10] An CH Kim MS Yoo NJ Park SW Lee SH. Mutational and Expressional Analyses of ATG5, an Autophagy-Related Gene, in Gastrointestinal Cancers. Pathol Res Pract (2011) 207(7):433–7. doi: 10.1016/j.prp.2011.05.002 21664058

[11] Grimm WA Messer JS Murphy SF Nero T Lodolce JP Weber CR. The Thr300Ala Variant in ATG16L1 Is Associated With Improved Survival in Human Colorectal Cancer and Enhanced Production of Type I Interferon. Gut (2016) 65(3):456–64. doi: 10.1136/gutjnl-2014-308735 PMC4789828 25645662

[12] Liang C Feng P Ku B Dotan I Canaani D Oh BH. Autophagic and Tumour Suppressor Activity of a Novel Beclin1-Binding Protein UVRAG. Nat Cell Biol (2006) 8(7):688–99. doi: 10.1038/ncb1426 16799551

[13] Foerster EG Mukherjee T Cabral-Fernandes L Rocha JDB Girardin SE Philpott DJ. How Autophagy Controls the Intestinal Epithelial Barrier. Autophagy (2021) 27:1–18. doi: 10.1080/15548627.2021.1909406 PMC8865220 33906557

[14] Lan SH Wu SY Zuchini R Lin XZ Su IJ Tsai TF. Autophagy Suppresses Tumorigenesis of Hepatitis B Virus-Associated Hepatocellular Carcinoma Through Degradation of microRNA-224. Hepatology (2014) 59(2):505–17. doi: 10.1002/hep.26659 PMC4298796 23913306

[15] Chu CA Lee CT Lee JC Wang YW Huang CT Lan SH. MiR-338-5p Promotes Metastasis of Colorectal Cancer by Inhibition of Phosphatidylinositol 3-Kinase, Catalytic Subunit Type 3-Mediated Autophagy Pathway. EBioMedicine (2019) 43:270–81. doi: 10.1016/j.ebiom.2019.04.010 PMC6557806 30982765

[16] Zhou K Liu M Cao Y. New Insight Into microRNA Functions in Cancer: Oncogene-microRNA-Tumor Suppressor Gene Network. Front Mol Biosci (2017) 4:46. doi: 10.3389/fmolb.2017.00046 28736730 PMC5500619

[17] Frankel LB Lund AH. MicroRNA Regulation of Autophagy [Research Support, Non-U.S. Gov'tReview]. Carcinogenesis (2012) 33(11):2018–25. doi: 10.1093/carcin/bgs266 22902544

[18] Tseng YS Tzeng CC Huang CY Chen PH Chiu AW Hsu PY. Aurora-A Overexpression Associates With Ha-Ras Codon-12 Mutation and Blackfoot Disease Endemic Area in Bladder Cancer. Cancer Lett. (2006) 241(1):93–101. doi: 10.1016/j.canlet.2005.10.014. S0304-3835(05)00909-2. 16338065

[19] Wu SY Lan SH Cheng DE Chen WK Shen CH Lee YR. Ras-Related Tumorigenesis Is Suppressed by BNIP3-Mediated Autophagy Through Inhibition of Cell Proliferation. Neoplasia (2011) 13(12):1171–82. doi: 10.1593/neo.11888 PMC3257192 22241963

[20] Li L Liu H Du L Xi P Wang Q Li Y. MiR-449a Suppresses LDHA-Mediated Glycolysis to Enhance the Sensitivity of Non-Small Cell Lung Cancer Cells to Ionizing Radiation. Oncol Res (2017) 7;26(4):547–56. doi: 10.3727/096504017X15016337254605 PMC7844793 28800787

[21] Sun X Liu S Chen P Fu D Hou Y Hu J. miR-449a Inhibits Colorectal Cancer Progression by Targeting SATB2. Oncotarget (2017) 8(60):100975–88. doi: 10.18632/oncotarget.10900 PMC5731849 29254139

[22] Feng Y Dong YW Song YN Xiao JH Guo XY Jiang WL. MicroRNA449a Is a Potential Predictor of Colitisassociated Colorectal Cancer Progression. Oncol Rep (2018) 40(3):1684–94. doi: 10.3892/or.2018.6566 30015944

[23] Fu D Chen Y Xu D. Circulating miR-449a Predicts Survival Outcome for Colorectal Cancer Following Curative Resection: An Observational Study. Med (Baltimore) (2021) 100(15):e25022. doi: 10.1097/MD.0000000000025022 PMC8052019 33847612

[24] Balgi AD Fonseca BD Donohue E Tsang TC Lajoie P Proud CG. Screen for Chemical Modulators of Autophagy Reveals Novel Therapeutic Inhibitors of Mtorc1 Signaling. PloS One (2009) 4(9):e7124. doi: 10.1371/journal.pone.0007124 19771169 PMC2742736

[25] Li X Wan T Li Y. Role of FoxO1 in Regulating Autophagy in Type 2 Diabetes Mellitus (Review). Exp Ther Med (2021) 22(1):707. doi: 10.3892/etm.2021.10139 34007316 PMC8120662

[26] Klotz LO Sanchez-Ramos C Prieto-Arroyo I Urbanek P Steinbrenner H Monsalve M. Redox Regulation of FoxO Transcription Factors. Redox Biol (2015) 6:51–72. doi: 10.1016/j.redox.2015.06.019 26184557 PMC4511623

[27] Chen J Halappanavar S Th' ng JP Li Q. Ubiquitin-Dependent Distribution of the Transcriptional Coactivator P300 in Cytoplasmic Inclusion Bodies. Epigenetics (2007) 2(2):92–9. doi: 10.4161/epi.2.2.4326 17965593

[28] Komatsu M Waguri S Koike M Sou YS Ueno T Hara T. Homeostatic Levels of P62 Control Cytoplasmic Inclusion Body Formation in Autophagy-Deficient Mice. Cell (2007) 131(6):1149–63. doi: 10.1016/j.cell.2007.10.035 18083104

[29] Noonan EJ Place RF Pookot D Basak S Whitson JM Hirata H. miR-449a Targets HDAC-1 and Induces Growth Arrest in Prostate Cancer [Research Support, N.I.H., Extramural Research Support, U.S. Gov't, Non-P.H.S.]. Oncogene (2009) 28(14):1714–24. doi: 10.1038/onc.2009.19 19252524

[30] Bou Kheir T Futoma-Kazmierczak E Jacobsen A Krogh A Bardram L Hother C. miR-449 Inhibits Cell Proliferation and Is Down-Regulated in Gastric Cancer [Research Support, Non-U.S. Gov't]. Mol Cancer (2011) 10:29. doi: 10.1186/1476-4598-10-29 21418558 PMC3070685

[31] Naderi T Mohammadi-Yeganeh S Mohammadi-Hezaveh N Hadavi R Gharehbaghian A Vazifeh-Shiran N. Investigating the Inhibitory Effect of miR-34a, miR-449a, miR-1827, and miR-106b on Target Genes Including NOTCH1, C-Myc, and CCND1 in Human T Cell Acute Lymphoblastic Leukemia Clinical Samples and Cell Line. Iran J Basic Med Sci (2020) 23(3):376–82. doi: 10.22038/IJBMS.2019.40695.9615 PMC7229500 32440325

[32] Wu X Han Y Liu F Ruan L. Downregulations of miR-449a and miR-145-5p Act as Prognostic Biomarkers for Endometrial Cancer. J Comput Biol (2020) 27(5):834–44. doi: 10.1089/cmb.2019.0215 31513434

[33] Chandrashekar DS Bashel B Balasubramanya SAH Creighton CJ Ponce-Rodriguez I Chakravarthi B. UALCAN: A Portal for Facilitating Tumor Subgroup Gene Expression and Survival Analyses. Neoplasia (2017) 19(8):649–58. doi: 10.1016/j.neo.2017.05.002 PMC5516091 28732212

[34] Niki M Nakajima K Ishikawa D Nishida J Ishifune C Tsukumo SI. MicroRNA-449a Deficiency Promotes Colon Carcinogenesis. Sci Rep (2017) 7(1):10696. doi: 10.1038/s41598-017-10500-0 28878284 PMC5587792

[35] Ozsolak F Poling LL Wang Z Liu H Liu XS Roeder RG. Chromatin Structure Analyses Identify miRNA Promoters [Research Support, N.I.H., Extramural Research Support, Non-U.S. Gov't]. Genes Dev (2008) 22(22):3172–83. doi: 10.1101/gad.1706508 PMC2593607 19056895

[36] Zhang X Yang S Chen J Su Z. Unraveling the Regulation of Hepatic Gluconeogenesis. Front Endocrinol (Lausanne) (2018) 9:802. doi: 10.3389/fendo.2018.00802 30733709 PMC6353800

[37] Zhao Y Yang J Liao W Liu X Zhang H Wang S. Cytosolic FoxO1 Is Essential for the Induction of Autophagy and Tumour Suppressor Activity [Research Support, Non-U.S. Gov't]. Nat Cell Biol (2010) 12(7):665–75. doi: 10.1038/ncb2069 20543840

[38] Jiramongkol Y Lam EW. FOXO Transcription Factor Family in Cancer and Metastasis. Cancer Metastasis Rev (2020) 39(3):681–709. doi: 10.1007/s10555-020-09883-w 32372224 PMC7497309

[39] Ishihama K Yamakawa M Semba S Takeda H Kawata S Kimura S. Expression of HDAC1 and CBP/p300 in Human Colorectal Carcinomas. J Clin Pathol. (2007) 60(11):1205–10. doi: 10.1136/jcp.2005.029165 PMC2095491 17720775

[40] Jacomin AC Samavedam S Promponas V Nezis IP. iLIR Database: A Web Resource for LIR Motif-Containing Proteins in Eukaryotes. Autophagy (2016) 12(10):1945–53. doi: 10.1080/15548627.2016.1207016 PMC5079668 27484196

[41] Montalto FI De Amicis F. Cyclin D1 in Cancer: A Molecular Connection for Cell Cycle Control, Adhesion and Invasion in Tumor and Stroma. Cells (2020) 9(12):2648–62. doi: 10.3390/cells9122648 PMC7763888 33317149

[42] Wu SY Lan SH Wu SR Chiu YC Lin XZ Su IJ. Hepatocellular Carcinoma-Related Cyclin D1 Is Selectively Regulated by Autophagy Degradation System. Hepatology (2018) 68(1):141–54. doi: 10.1002/hep.29781 PMC6055810 29328502

[43] Wu SY Lan SH Liu HS. Degradative Autophagy Selectively Regulates CCND1 (Cyclin D1) and MIR224, Two Oncogenic Factors Involved in Hepatocellular Carcinoma Tumorigenesis. Autophagy (2019) 15(4):729–30. doi: 10.1080/15548627.2019.1569918 PMC6526824 30646811

[44] Li Y Guessous F Johnson EB Eberhart CG Li XN Shu Q. Functional and Molecular Interactions Between the HGF/c-Met Pathway and C-Myc in Large-Cell Medulloblastoma [Research Support, N.I.H., Extramural Research Support, Non-U.S. Gov't]. Lab Invest; J Tech Methods Pathol. (2008) 88(2):98–111. doi: 10.1038/labinvest.3700702 18059365

[45] Lin AY Chua MS Choi YL Yeh W Kim YH Azzi R. Comparative Profiling of Primary Colorectal Carcinomas and Liver Metastases Identifies LEF1 as a Prognostic Biomarker [Comparative Study Research Support, N.I.H., Extramural Research Support, Non-U.S. Gov't]. PloS One (2011) 6(2):e16636. doi: 10.1371/journal.pone.0016636 21383983 PMC3044708

[46] Deng X Cheng J Zhan N Chen J Zhan Y Ni Y. Analysis of Differentially Expressed Proteins Involved in miR-449a for Hepatocellular Carcinoma by iTRAQ Proteomics. Clin Lab (2021) 67(3):738–46. doi: 10.7754/Clin.Lab.2020.200628 33739042

[47] Olaru AV Yamanaka S Vazquez C Mori Y Cheng Y Abraham JM. MicroRNA-224 Negatively Regulates P21 Expression During Late Neoplastic Progression in Inflammatory Bowel Disease [Research Support, N.I.H., Extramural Research Support, Non-U.S. Gov't]. Inflamm Bowel Dis (2013) 19(3):471–80. doi: 10.1097/MIB.0b013e31827e78eb PMC4259288 23399735

