# Supplementary material for: Autophagy Upregulates miR-449a Expression to Suppress Progression of Colorectal Cancer
Source: Front Oncol. 2021 Oct 19;11:738144. doi: 10.3389/fonc.2021.738144 (PMC8560741; doi:10.3389/fonc.2021.738144)
Supplement: Supplementary file 1 [file DataSheet_1.pdf]

# Supplementary Material

## 1. Materials and Methods

### 1.1 *Immunoblotting*

Total protein was collected from the cells after various treatments. For Western blotting, a previously described procedure was applied (Wu et al., 2018). The following antibodies were used: The primary antibodies used to detect specific protein were β-actin (Sigma), LC3 (MBL), p300 (Santa Cruz), FoxO1 (Cell Signaling), AC-FKHR (Santa Cruz), and LEF-1 (Abcam).

### 1.2 *Immunofluorescent staining*

The treated cells were fixed in 3.7% formaldehyde for 30 min. After washing with phosphate-buffered saline (PBS), the cells were incubated for 30 min in 0.1% Triton X-100 in PBS. Anti-LC3 antibody (MBL, PM036), anti-FoxO1 antibody (Cell Signaling, C29H4), and anti-p300 antibody (Santa Cruz, sc-585) were added to the plate at 4°C overnight. Hoechst (5 mg/ml; Sigma) at a dilution of 1:100 in PBS was used to stain the nuclei. The fluorescent change of the cells was determined under a multi-photon confocal microscope (Olympus, FV1000MPE, Tokyo, Japan).

### 1.3 *Plasmid construction*

The restriction enzymes of *Sac* I and *Xho* I were used to digest the 5'end promoter region of miR-449a (the sequence of 2000 (2k), 1000 (1k), and 500 (0.5k) base pairs (bp) in front of the miR-449a gene), and the fragments were ligated into the pGL3-basic reporter vector.

### 1.4 *BrdU incorporation assay*

The miRNA-transfected cells ($5\times10^5$) were seeded in a six-well plate and treated with Amiodarone for 48 h. The cells were grown in bromodeoxyuridine (BrdU; 0.02 g/ml; Sigma, B5002) containing medium for 30 min, fixed in acidic ethanol at -20°C for 10 min and then incubated in 2N HCl for 10 min. Anti-BrdU polyclonal antibody (GE Healthcare, RPN202) at a dilution of 1:100 was added to the well at 4˚C overnight. The primary antibody was detected by FITC-conjugated goat anti-Mouse IgG under a fluorescent microscope (Olympus, DP 72, PA, USA).

### 1.5 *Transfection of miRNA and protein*

The cells ($2\times10^5$/well) were placed in a six-well plate and transfected with miR-449a (100 nM) (Pre-miR™ miRNA Precursor; Applied Biosystems), anti-miR-449a (100 nM) (Anti-miR™ miRNA Inhibitors; Applied Biosystems), and pcDNA3/Flag-FoxO1 (4 μg) by Lipofectamine 2000™ following the manufacturer's instructions (Invitrogen). Plasmid pFlag-CMV2 was used as

a vector control (Invitrogen). The small RNA controls were pre-miRNA negative control (Applied Biosystems) and anti-miRNA negative control (Applied Biosystems).

### 1.6 *Cell migration and invasion assay*

Transwell® containing an 8 μm pore diameter permeable insert (Corning Costar Corp. MA, USA) was used together with a 24-well plate. In the invasion assay, the transwell insert was precoated with the matrigel (1 mg/ml, BD Biosciences, MA, USA). Serum-free medium was added in the lower chamber. Transfected cells ($5 \times 10^5$/100 μl) seeded in the upper chamber were maintained at 37°C. After incubation for 48 h (for migration) or 72 h (for invasion), the cells remaining on the upper side of the chamber were removed with cotton swabs. The cells that had migrated to the lower side of the chamber were fixed with 1% formaldehyde (Sigma) and stained with 0.1% crystal violet. Migrated cells in the lower chamber were photographed and counted under a light microscope. The cells were counted under three random fields.

Supplementary Figures and Tables

## 2.1 Supplementary Table 1

**Log ratio ($Log_2RQ$) of down-regulated miRNAs in MEF-*Atg5(-/-)*-Ras and MEF-*Atg5(+/+)*-Ras cells and derived tumors**

| miRNA | Cells | Tumors | Regulation |
|---|---|---|---|
| mmu-miR-449a | 2.16§ | 5.01§ | Down |
| mmu-miR-129-3p | 5.71 | 2.76 | Down |
| mmu-miR-129-5p | 4.45 | 2.43 | Down |
| mmu-miR-296-3p | 2.81 | 3.17 | Down |
| mmu-miR-296-5p | 3.98 | 2.88 | Down |

- MEF-*Atg5 (+/+)*-Ras and MEF-*Atg5 (-/-)*-Ras: *Atg5* gene wild-type or knockout in mouse embryonic fibroblasts with *H-ras* overexpression.
- Tumors: Above cells were inoculated in NOD/SCID mice by subcutaneous injection and tumors were collected at 21 days after injection.
- §Mean fold change of miRNAs in MEF-*Atg5(-/-)*-Ras cells and tumors compared with MEF-*Atg5(+/+)*-Ras cells and tumors ($Log_2RQ$)
- $Log_2(RQ)$ corresponds to the Log Ratio value in MEF-*Atg5(-/-)*-Ras and MEF-*Atg5(+/+)*-Ras cells and tumors.
- RQ stands for relative quantification by real-time PCR analysis.

## 2.2 Supplementary Table 2

**The expression levels of five miRNAs under various autophagic induction and inhibition conditions**

| Treatment \ MiRNAs | miR-449a | miR-129-3-p | miR-129-5-p | miR-296-3-p | miR-296-5-p |
|---|---|---|---|---|---|
| IPTG* | + | - | - | + | - |
| Rapamycin | + | - | + | + | - |
| HBSS | + | - | - | - | - |
| sh-Atg5 | - | - | - | - | - |

**+: Significant up-regulation; - : Significant down-regulation**

- IPTG: Isopropyl β-D-1-thiogalactopyranoside; HBSS: Hanks' Balanced Salt Solution; sh-Atg5: Lentiviral sh-RNA for Atg5

* Ha-*ras* oncogene overexpression was induced by IPTG treatment.

## 2.3 Supplementary Table 3

**Characteristics of group I 56 CRC patient specimens**

| Parameter | Number |
|---|---|
| CRC specimens | 56 |
| Gender (Male/Female) | 31/25 |
| AJCC (TNM) Stage (I/II/III/IV) | 17/11/16/12 |
| Dukes Stage (A/B/C/D) | 15/13/16/12 |
| Metastasis (No/Yes) | 28/28 |
| Recurrence (No/Yes) | 42/14 |

## 2.4 Supplementary Table 4

**Characteristics of group II 51CRC patient specimens from tissue array**

| Parameter | Number |
|---|---|
| CRC specimens | 51 |
| Gender (Male/Female) | 22/29 |
| Stage (A/B/C/D) | 6/19/21/5 |
| Metastasis (No/Yes) | 47/4 |
| Recurrence (No/Yes) | 38/13 |

## 2.5 Supplementary Table 5

**Characteristics of CRC patient specimens from the tissue array**

| Parameter | Number |
|---|---|
| CRC specimens | 18 |
| Stage (A/B/C/D) | 1/7/6/4 |
| Metastasis (No/Yes) | 8/10 |
| Survival (No/Yes) | 4/14 |

**2.6 Supplementary Figure 1. The expression of miR-449a in seven human cancer cell lines.**

The expression of miR-449a was determined by real-time RT–PCR in prostate (PC3 cells), gastric (AGS cells), liver (Huh7 and Hep3B cells), breast (MCF-7 cells), lung (A549 cells), and colorectal (SW480) cancers. U54 was used as an endogenous control to normalize miR-449a expression.

**2.7 Supplementary Figure 2. ROC analysis of clinical CRC patient specimens.**

(A) ROC analysis to discriminate miR-449a expression in 56 CRC patient specimens grouped as stage A and stages B+C+D. (B) ROC analysis of miR-449a expression grouped as non-metastasis and metastasis patients. The AUC value was calculated to evaluate the specificity and sensitivity of miR-449a expression. The *p* values were calculated by paired t-test.

**2.8 Supplementary Figure 3. Beclin 1 expression analysis by Clinical Proteomic Tumor Analysis Consortium**

The analysis of BECN1 expression in normal tissue and primary tumor groups from Clinical Proteomic Tumor Analysis Consortium (CPTAC). The data is obtained from UALCAN website http://ualcan.path.uab.edu/index.html (Chandrashekar et al., 2017).

**2.9 Supplementary Figure 4. The expression of miR-449a is regulated by FoxO1.**

(A) CDC20B mRNA expression was evaluated after amiodarone (10 μM) treatment for 48 h by real-time PCR using specific primer sets. (B) The cells were treated with amiodarone (10 μM) in the presence or absence of FoxO1 inhibitor AS1842856 (40 nM) for 48 h. The activity of miR-449 reporter plasmid was determined by luciferase assay.

**2.10 Supplementary Figure 5. The effect of miR-449a expression on target gene LEF-1 and cyclin D1 RNA and protein expression in CRC SW480 cells.**

(A) The SW480 and SW620 cells were transiently transfected with 100 nM of miR-449a, anti-miR-449a, or scramble control (anti-N.C.) for 48 h using Lipofectamine™ 2000 reagent. The expression levels of LEF-1 and cyclin D1 proteins were determined by immunoblotting using specific antibodies. For SW480 cells, the relative LEF-1 RNA expression in (B) and cyclin D1 RNA expression in (C) was determined by real-time PCR. β-actin was used as an endogenous control to normalize the expression of genes. (D) SW480, SW620, HCT116, and HT29 cells were treated with amiodarone for 48 h and the protein expression of LC3, LEF-1, and/or cyclin D1 were determined by immunoblotting using specific antibodies. β-actin was used as the internal control

**2.11 Supplementary Figure 6. The effect of amiodarone on SW480 cell tumorigenesis.**

The SW480 cells were treated with amiodarone. (A) Cell number was counted under a light microscope for four days after amiodarone treatment. (B) Cell proliferation was determined by BrdU (0.01 g/ml) incorporation assay for 30 min after the cells were treated with amiodarone. (C) A total of $2\times10^4$ cells treated with amiodarone were plated on the soft agar. The colonies were taken 14 days after plating. The bottom shows the quantification of colonies in the plates. Scale bar= 1

mm. (D) Cell migration assay was conducted with or without amiodarone treatment. The number of migrated cells was counted after 48 h. (E) Invasion assay was conducted after amiodarone treatment. The number of invasive cells was counted after 72 h. The quantifications represent the results of three independent experiments. Scale bar= 100 µm. The *P*-values were calculated by Student's *t*-test.

**2.12 Supplementary Figure 7. Clinical relevance of FoxO1, LEF-1 and cyclin D1 in CRC patient specimens on the tissue array.**

Clinical information of the 18 paired CRC specimens in the tissues array is presented in Supplementary Table 5. IHC staining is used to detect the expression of FoxO1, LEF-1 and cyclin D1 in the CRC specimens on the tissue array plate. FoxO1 was detected in the nuclei of tumor cells. Histoquest software was used to quantify the protein expression level. The p-values were calculated by paired t-test. Scale bar represents 20 µm.

**2.13 Supplementary Figure 8. TCGA CRC dataset analysis for gene expression and overall survival rate of Beclin 1, LEF-1, and cyclin D1 gene.**

(A) and (B) Graph representing the relative gene expression of cyclin D1 (CCND1) and LEF1 in normal tissue and primary tumor groups from TCGA dataset. (C) Survival curve shown for CRC patients using LEF-1 transcript levels. Patients based on the expression of LEF-1 transcript levels were divided into two groups. Survival of two CRC patient groups, LEF-1 high (red) and LEF-1 low (blue), is shown (*P*= .030).

**2.14 References**

Chandrashekar, D.S., Bashel, B., Balasubramanya, S.A.H., Creighton, C.J., Ponce-Rodriguez, I., Chakravarthi, B., et al. (2017). UALCAN: A Portal for Facilitating Tumor Subgroup Gene Expression and Survival Analyses. *Neoplasia* 19(8)**,** 649-658. doi: 10.1016/j.neo.2017.05.002.

Wu, S.Y., Lan, S.H., Wu, S.R., Chiu, Y.C., Lin, X.Z., Su, I.J., et al. (2018). Hepatocellular carcinoma-related cyclin D1 is selectively regulated by autophagy degradation system. *Hepatology* 68(1)**,** 141-154. doi: 10.1002/hep.29781.

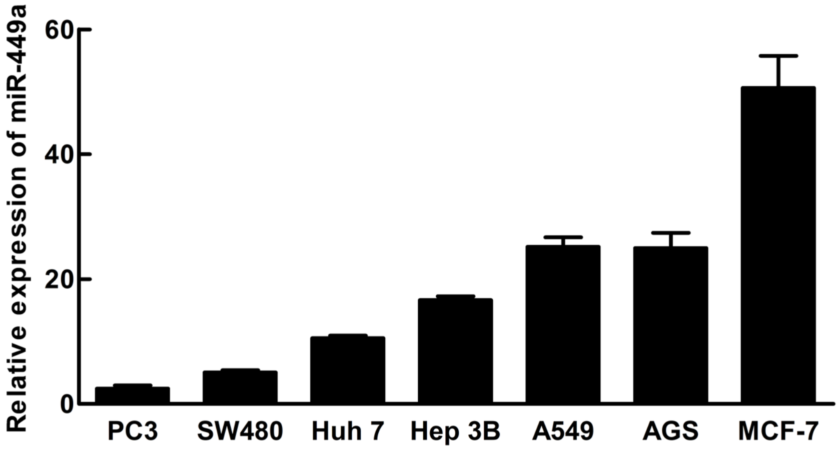


## Supplementary Figure 1

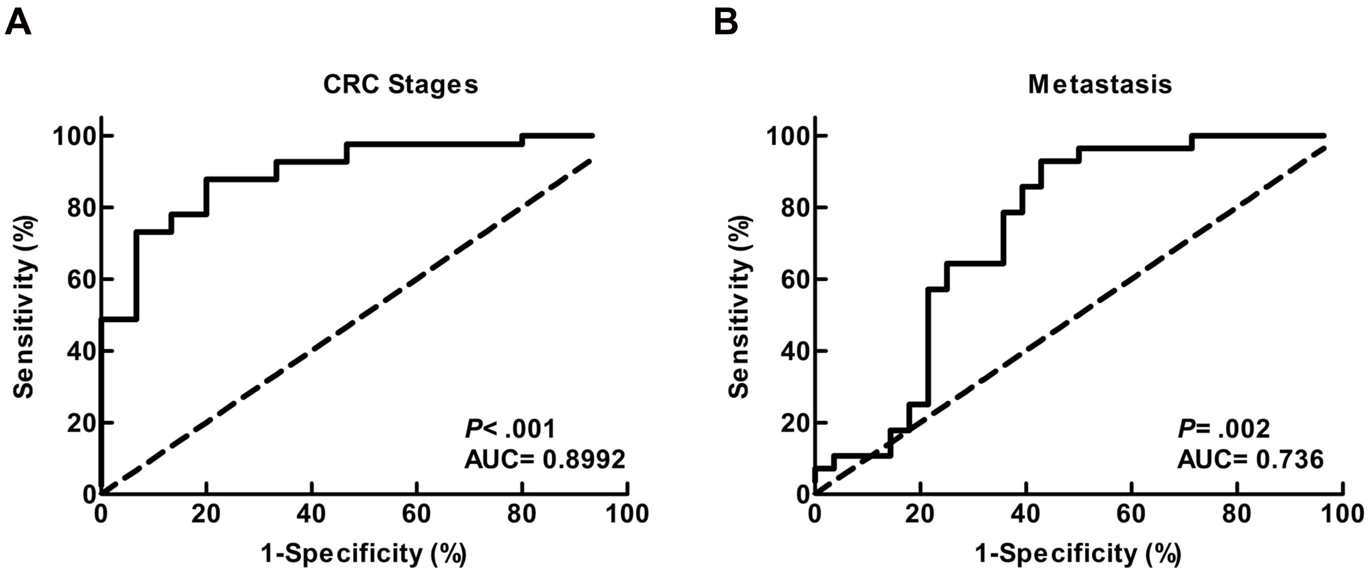


**Supplementary Figure 2**

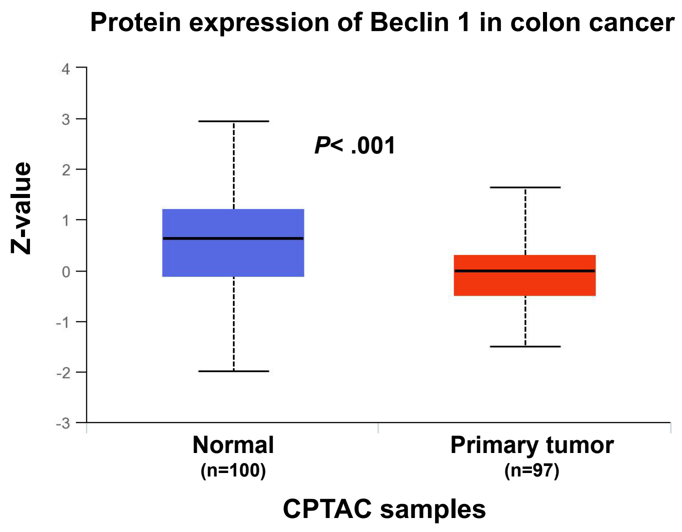


# Supplementary Figure 3

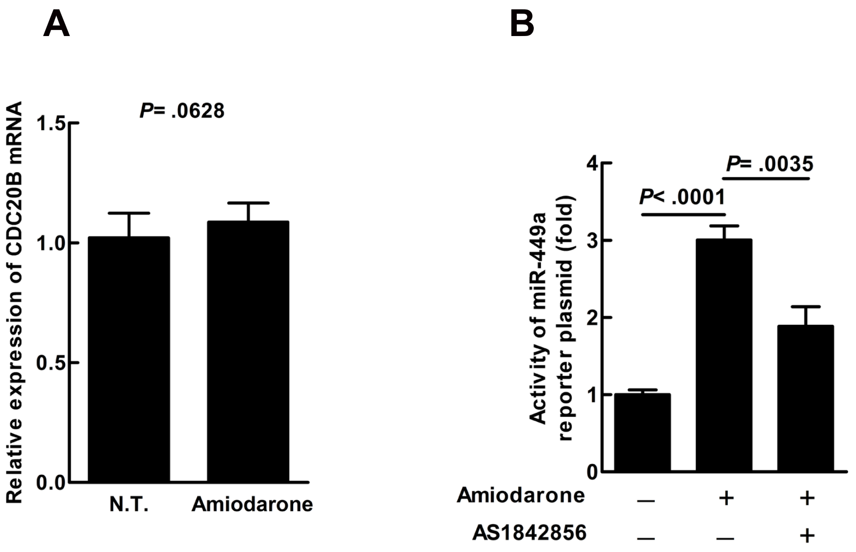


# Supplementary Figure 4

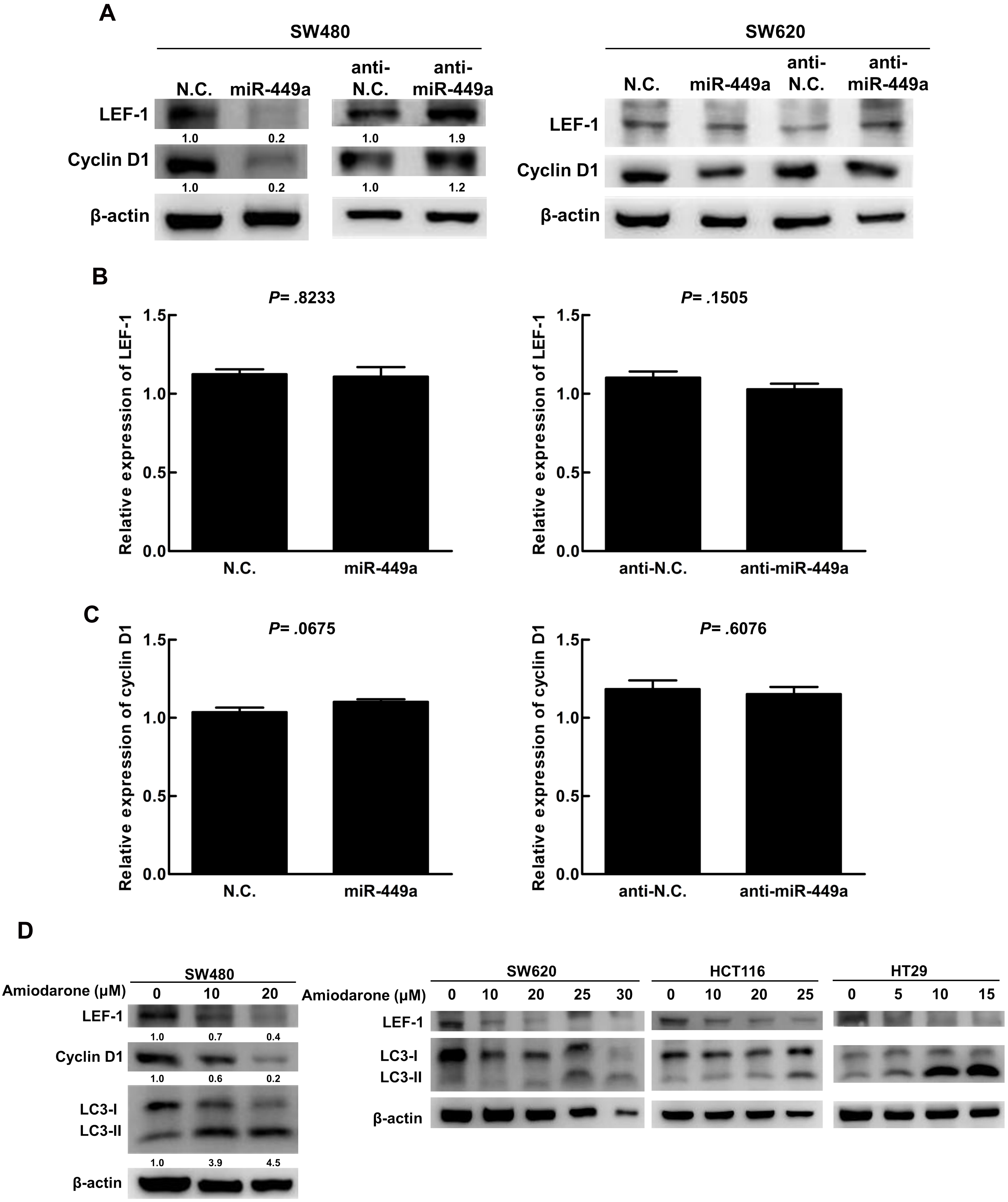


Supplementary Figure 5

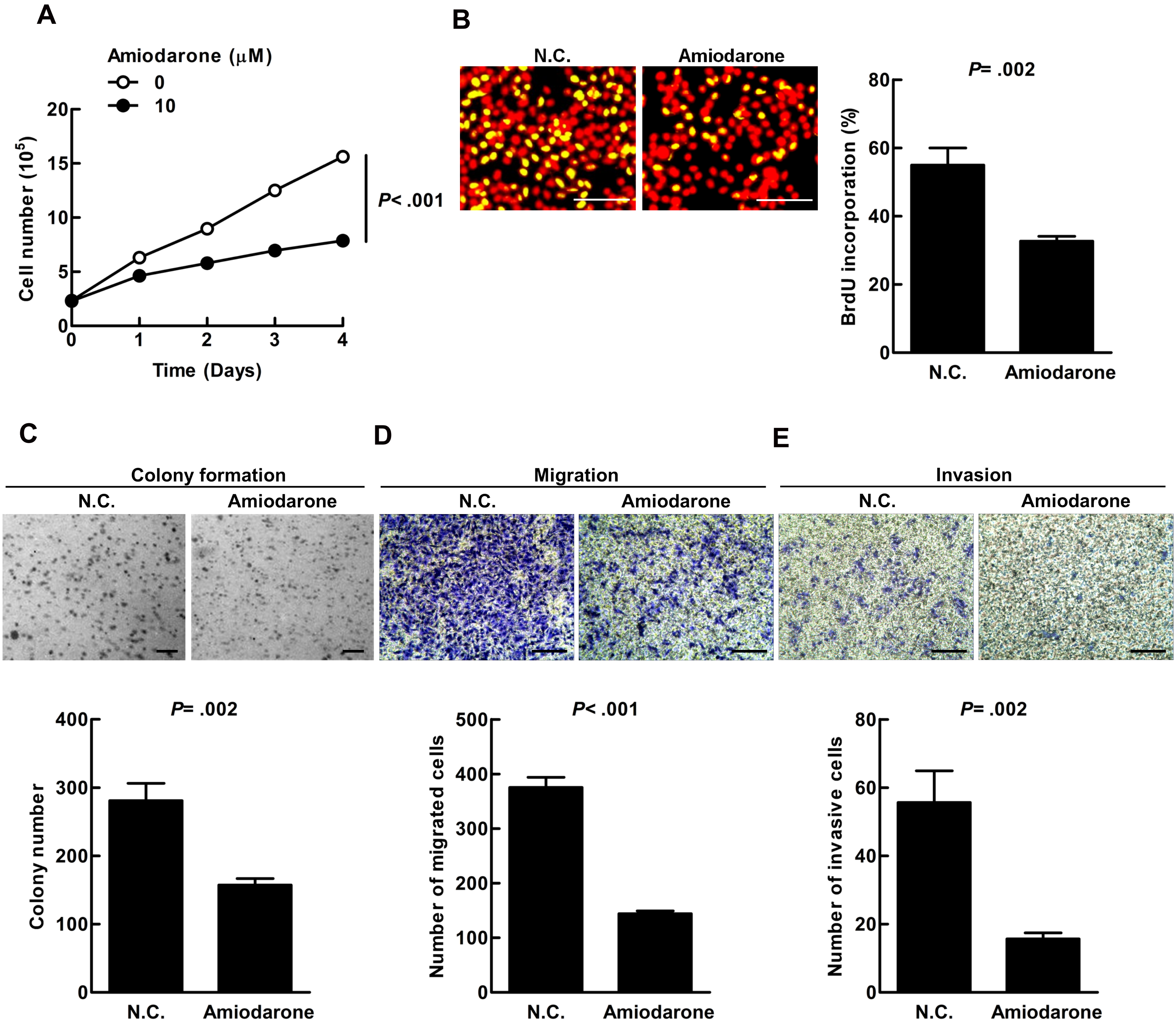


**Supplementary Figure 6**

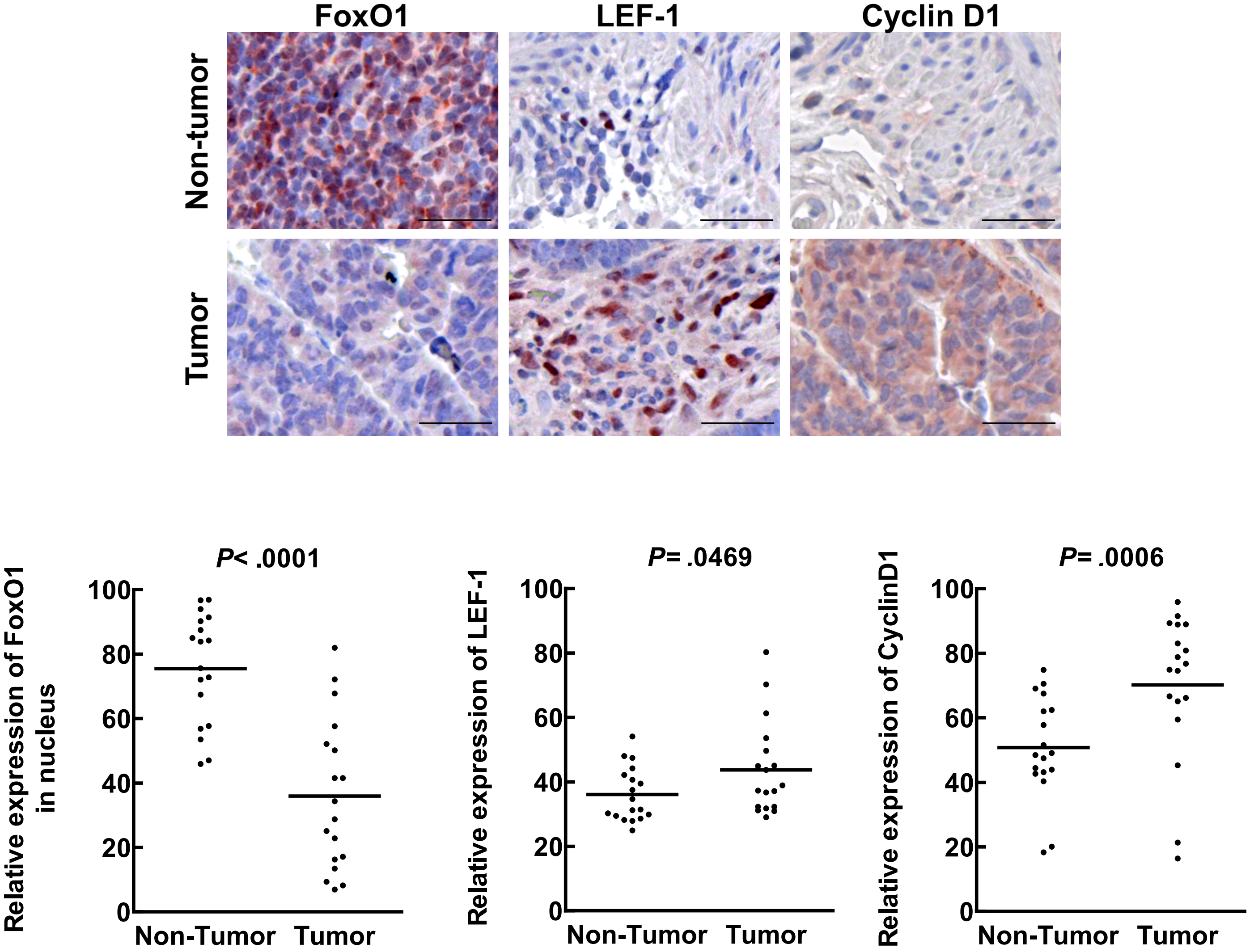


**Supplementary Figure 7**

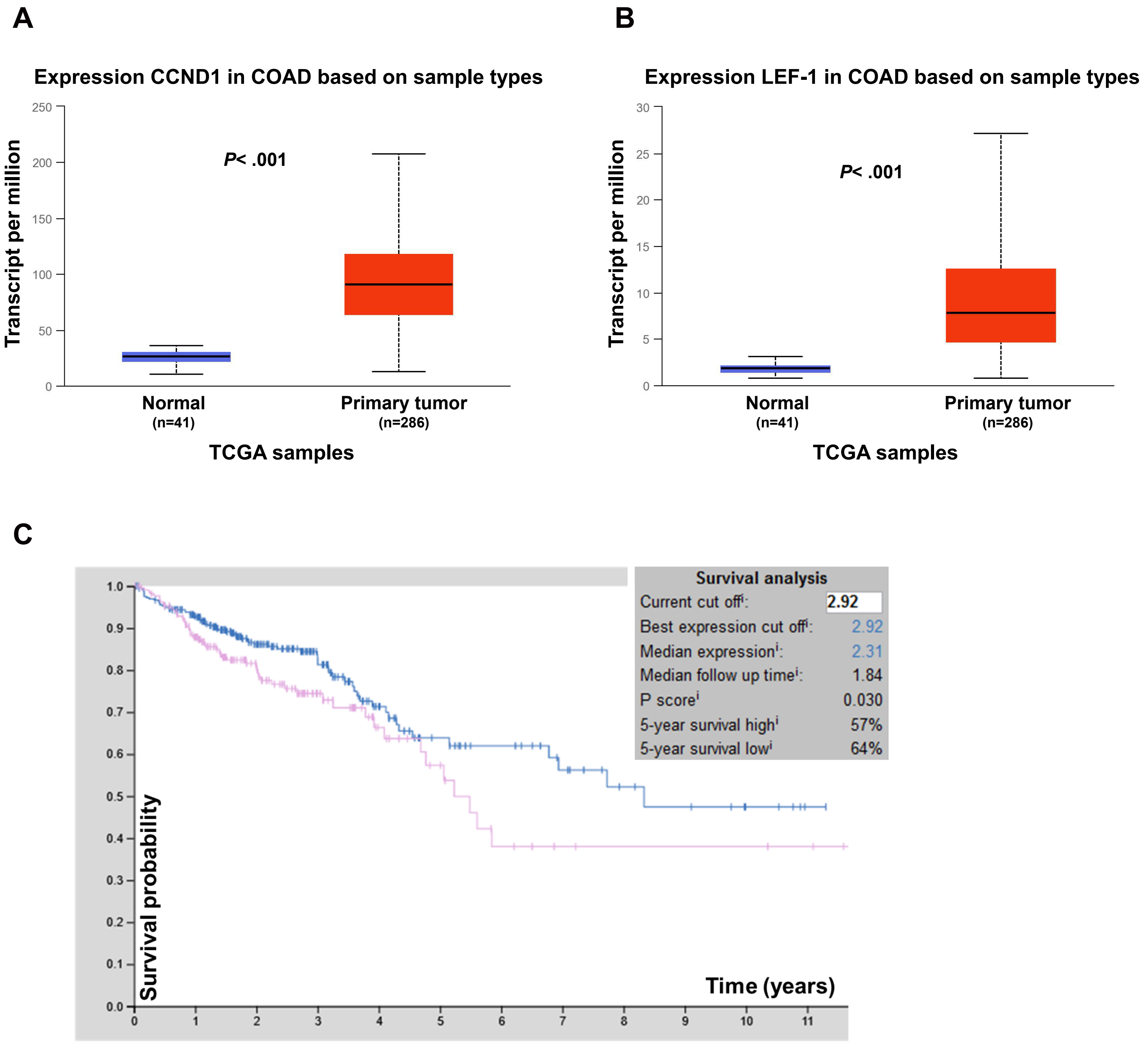


# Supplementary Figure 8
